# Clinicopathological characteristics and prognostic factors in axial chondroblastomas: a retrospective analysis of 61 cases and comparison with extra-axial chondroblastomas

**DOI:** 10.1186/s12957-023-03063-0

**Published:** 2023-06-21

**Authors:** Bo-Wen Zheng, Bo-Yv Zheng, Hua-Qing Niu, Ming-Xiang Zou, Hai-Lin Wu, Ming Wang, Xue-Lin Li

**Affiliations:** 1 Department of Spine Surgery, The First Affiliated Hospital, Hengyang Medical School, University of South China, Hengyang, 421001 China; 2 Musculoskeletal Tumor Center, People’s Hospital, Peking University; Beijing Key Laboratory of Musculoskeletal Tumor, Beijing, China; 3 Department of Spine Surgery, The Second Xiangya Hospital, Central South University, Changsha, 410011 China; 4 Department of Orthopedics Surgery, General Hospital of the Central Theater Command, Wuhan, 430061 China; 5 Department of Ophthalmology, The Second Affiliated Hospital of Zhengzhou University, Zhengzhou, 450014 China; 6 Department of Spine Surgery, Hengyang Medical School, The First Affiliated Hospital, University of South China, 69 Chuanshan Road, Hengyang, Hunan 421001 China

**Keywords:** Axial chondroblastomas, Extra-axial chondroblastomas, Prognostic factors, Survival, Comparative study

## Abstract

**Background:**

A comprehensive understanding of the clinical characteristics and prognostic factors associated with axial chondroblastoma (ACB) is still lacking. This study aimed to understand the clinical characteristics and prognostic factors of axial chondroblastoma (ACB) and compare them with extra-axial chondroblastoma (EACB).

**Methods:**

A retrospective review of our institution’s local database was conducted, encompassing a total of 132 CB patients, of which 61 were diagnosed with ACB and 71 with EACB. Immunohistochemistry was employed to evaluate the expression levels of vimentin, S100, and cytokeratin.

**Results:**

ACB and EACB shared similar characteristics, with the exception of advanced age, tumor size, elevated Vim expression, incidence of surrounding tissue invasion, and postoperative sensory or motor dysfunction. While wide resection and absence of surrounding tissue invasion consistently showed a favorable association with survival in both ACB and EACB cohorts during univariate analysis, most parameters exhibited differential prognostic significance between the two groups. Notably, the significant prognostic factors for local recurrence-free survival in the ACB cohort included the type of resection and the presence of chicken-wire calcification. In the multivariate analysis of overall survival, the type of resection emerged as a significant predictor in the ACB cohort, whereas in the EACB group, the type of resection and the occurrence of postoperative sensory or motor dysfunction were predictive of overall survival.

**Conclusion:**

There may exist distinct biological behaviors between ACB and EACB, thereby providing valuable insights into the prognostic characteristics of ACB patients and contributing to enhanced outcome prediction in this particular patient population.

**Supplementary Information:**

The online version contains supplementary material available at 10.1186/s12957-023-03063-0.

## Introduction

Chondroblastoma (CB) is an uncommon neoplasm originating from the cartilage, characterized by its locally infiltrative growth patterns primarily observed in the epiphysis of long bones, comprising less than 1% of all bone tumors [1]. The current treatment approach for CB revolves around achieving complete excision of the tumor. However, due to its locally invasive nature and the potential proximity to critical neurovascular structures, achieving extensive complete resection may pose challenges during surgical intervention. Furthermore, conventional chemotherapy has proven ineffective in treating CB patients, and radiotherapy may even promote malignant transformation of the disease [2]. Consequently, the recurrence rate following surgery remains high in CB patients, significantly impacting their long-term quality of life and overall survival.

CB predominantly occurs in the extra-axial skeletal regions, with the metaphysis of long bones being the most common location [1]. Existing research on CB has primarily concentrated on its occurrence in the bones of the extremities. Previous investigations have unveiled the impact of CB’s biological behavior on the clinical outcomes of patients, particularly noting a higher susceptibility to recurrence following surgical intervention for tumors situated in the proximal pelvis and humerus [3, 4]. Furthermore, patient age and the presence of cyst formation have been identified as factors associated with CB recurrence [4–6]. These significant findings not only contribute valuable insights for prognostic risk stratification of extra-axial CB (EACB) but also pave the way for the development of novel treatment approaches.

In comparison with EACB, axial chondroblastomas (ACB) are considerably less common. Cranial CB, for instance, accounts for less than 2% of all CB patients and exhibits a notably higher recurrence rate in cases where postoperative residual lesions are present [7]. Similarly, spinal CB represents a mere 1.4% of all CB patients and demonstrates a greater tendency for recurrence when compared to CB occurring in the extremities [8]. Moreover, spinal CB displays a higher likelihood of recurrence and exhibits more aggressive tumor growth compared to its extremity counterpart [9–11]. While there have been reports of studies on ACB in the literature, most of them comprised single cases or small case series. Given the unfavorable prognosis associated with ACB, there is a pressing need for a systematic summary of prognostic factors and the development of a reasonable risk stratification approach. Such efforts would help optimize treatment planning and, consequently, improve the survival prognosis for patients. Therefore, the objective of this study is to provide a comprehensive analysis of a large sample of CB cases, summarizing the clinicopathological characteristics of ACB patients. We aim to identify the factors influencing local recurrence-free survival (LRFS) and overall survival (OS). Additionally, we conducted a comparative assessment of the clinicopathological attributes between ACB and EACB.

## Methods and materials

### Patients and tissue samples

A total of 132 patients were included in the study, comprising 61 patients with ACB and 71 patients with EACB. Recently, our research group communicated the patient characteristics [12]. Comprehensive patient and tumor characteristics, treatment history, and clinical outcome data were obtained from the patients’ medical records. The collected clinical information encompassed patient demographics (age and sex), clinical features (including duration of symptoms and preoperative/postoperative sensorimotor status), and treatment modalities (type of surgery and adjuvant radiotherapy). Preoperative magnetic resonance imaging was employed to evaluate the extent of tumor invasion into the surrounding tissues. The pathological diagnosis was independently confirmed by two neuropathologists based on examination of hematoxylin and eosin–stained sections and pathology findings, including the presence of secondary aneurysmal bone cyst (ABC) and chicken-wire calcification. The primary events of interest were LRFS and OS. LRFS was defined as the duration between tumor resection and the first recurrence, while OS referred to the time from surgical resection to the patient’s death from any cause [12]. The type of surgical resection was determined based on a previously reported method [13], distinguishing between wide resection (such as gross total or en bloc resection with negative margins) and non-wide resection (including intralesional or marginal resection).

### Immunohistochemistry staining and evaluation

Immunohistochemical analysis was conducted following the previously described protocol [12]. In brief, 4-μm paraffin-embedded tissue sections were deparaffinized in xylene and gradually rehydrated using a series of graded ethanol solutions, followed by rinsing in distilled water. Antigen retrieval and blocking were performed, after which the sections were incubated overnight at 4 °C with primary antibodies: anti-vimentin [Vim] (Abcam company) at a dilution of 1:400, anti-S100 (Abcam company) at a dilution of 1:100, and anti-cytokeratin [CK] (Abcam company) at a dilution of 1:20. Subsequently, the sections were treated with secondary biotinylated antibodies against rabbit or mouse immunoglobulins, followed by incubation with streptavidin-peroxidase conjugate (Auragene, Changsha, Hunan, China). Visualization was achieved using a solution of 3,3-diaminobenzidine, and the sections were counterstained with hematoxylin for preservation.

The immunohistochemical staining results were independently evaluated by two pathologists. Positive expression for S100, Vim, and CK was determined by the presence of yellow or brownish-yellow granules at the corresponding site. For each section, five randomly selected high-magnification fields were observed. The staining proportion in each field was quantified, and the mean value was calculated. The expression level of each immunohistochemical marker was assessed in the hematoxylin and eosin (HE) sections and categorized as absent (0), rare (1), moderate (2), or significant (3) according to a previously established method [14]. Tissue samples were considered negative if a score of 0–1 was observed and positive if the score was higher.

### Statistical analysis

The X-tile software version 3.6.1 (https://medicine.yale.edu/lab/rimm/research/software.aspx) was utilized to determine the threshold values for age, duration of symptoms, and tumor size in the survival analysis, with OS as the outcome parameter [15]. The threshold point represents the value that yields the minimum *p*-value from the corrected log-rank test [16]. Based on this threshold, patients were categorized into two subgroups: those with values less than or equal to the cutoff, and those with values greater than the cutoff. The cutoff point was specifically defined as the value that produced the minimum *p*-value from the log-rank test, which was duly corrected [17]. Statistical analyses were performed using the SPSS 26.0 software (SPSS, IBM, Armonk, NY). Descriptive statistics were presented as mean ± standard deviation, and comparisons were conducted using *t*-tests or ANOVA for continuous data. Categorical data were expressed as frequencies or proportions, and the chi-square test was employed for statistical analysis. Univariate Kaplan–Meier curves and log-rank tests were employed for survival analysis, exploring the associations between clinicopathological parameters and patient outcomes. A multivariate Cox proportional hazards model was employed to identify independent risk factors for LRFS and OS. Only variables that demonstrated statistical significance in the univariate survival analysis were included in the multivariate analysis. All hypothesis tests were two-sided, and a significance level of *P* < 0.05 was applied to determine statistical significance.

## Results

### Patient and tumor characteristics of CB patients

**Fig. 1:**
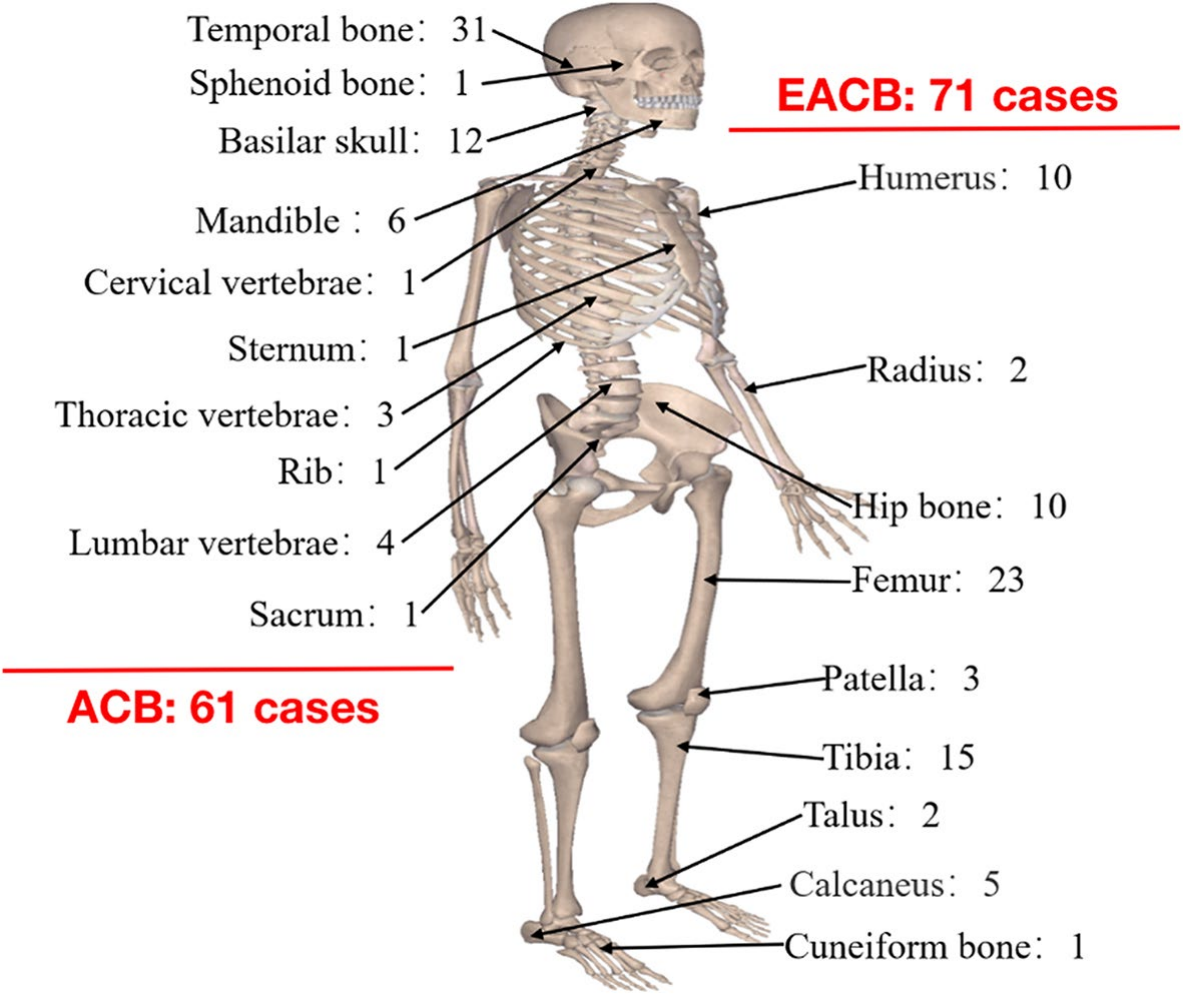
Distribution of the tumor site for 132 chondroblastoma patients

**Fig. 2:**
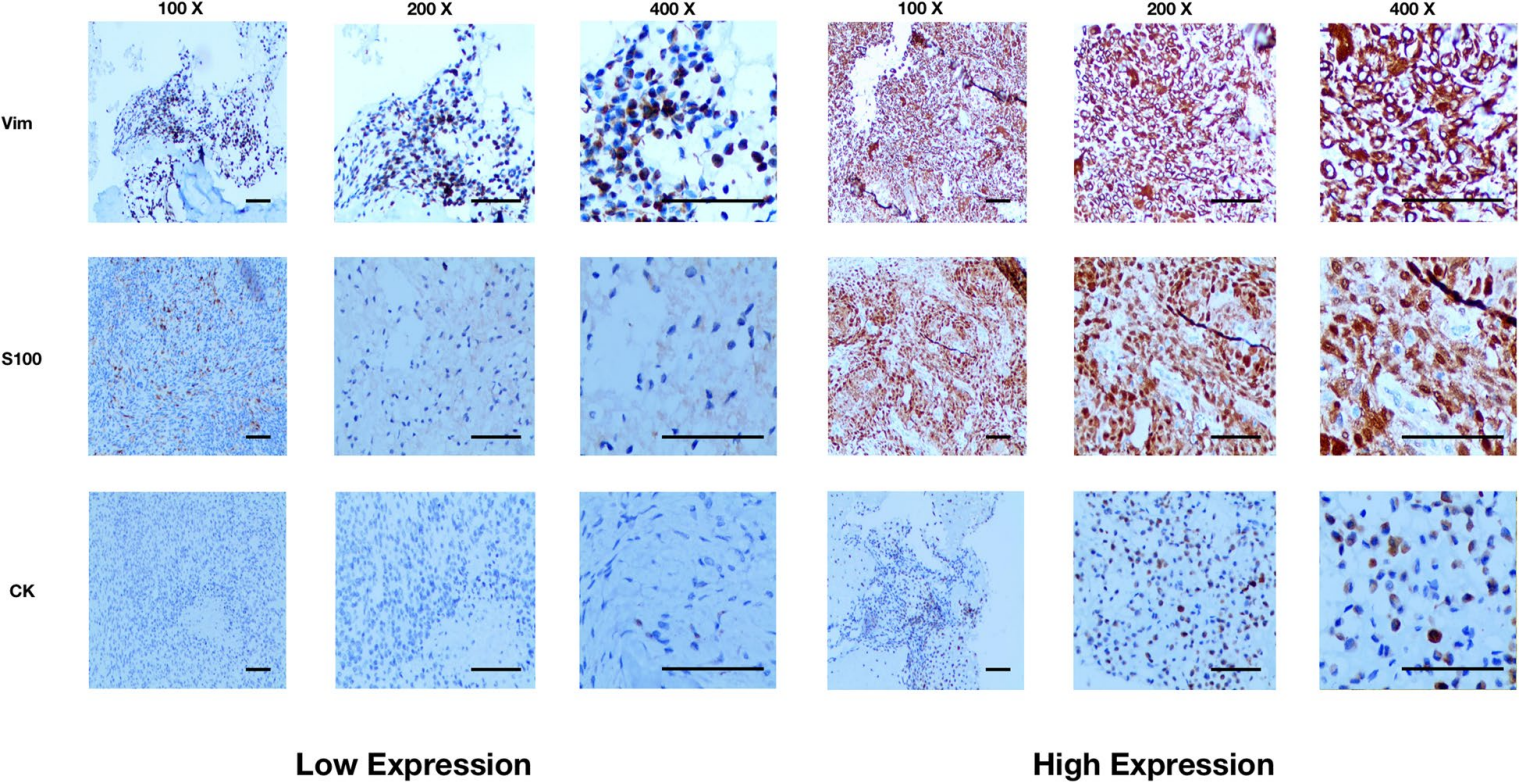
Representative images of immunohistochemical markers in chondroblastoma tissues. Vim, vimentin; CK, cytokeratin. Scale bar = 100 μm

A total of 132 patients diagnosed with CB were enrolled in this study, with 61 patients diagnosing ACB and 71 patients diagnosing EACB (Fig. 1). The patient characteristics are summarized in Table 1. Significant differences were observed between ACB and EACB patients in terms of age, tumor size, surrounding tissue invasion, preoperative sensory or motor dysfunction, and Vim expression (*P* < 0.001, *P* < 0.001, *P* < 0.001, *P* = 0.021, and *P* = 0.002, respectively). All patients underwent surgery, with 65 patients receiving wide resections and 67 patients receiving non-wide resections. None of the patients received chemotherapy. Postoperative adjuvant photon radiotherapy was administered to 34 patients. For the survival analysis of OS, age, tumor size, and symptom duration were used as cutoffs for subgroups in patients with ACB and EACB, as shown in Additional file 1: Supplemental Digital Content 1 and Supplemental Digital Content 2. Representative images of immunohistochemical markers can be seen in Fig. 2.

**Table 1:** Comparison of baseline characteristics between axial chondroblastoma and extra-axial chondroblastoma

Variable	Categories	All (*n*)	Axial (*n*)	Extra-axial (*n*)	*P*-value
Age (years)	Continuous	132 (29.2 ± 13.4)	61 (34.1 ± 14.6)	71 (24.9 ± 10.8)	** < 0.001**
Gender	Female	46	19	27	0.408
	Male	86	42	44	
Duration of symptoms (months)	Continuous	132 (8.3 ± 7.4)	61 (8.3 ± 6.7)	71 (8.3 ± 8.1)	0.955
Tumor size (in diameter, cm)	Continuous	132 (3.9 ± 1.8)	61 (5.3 ± 1.5)	71 (2.6 ± 0.9)	** < 0.001**
Type of resection	Wide	65	27	38	0.289
	Not wide	67	34	33	
Surrounding tissue invasion	No	59	12	47	** < 0.001**
	Yes	73	49	24	
Adjuvant radiotherapy	No	98	44	54	0.607
	Yes	34	17	17	
Preoperative sensory or motor dysfunction	No	97	39	58	**0.021**
	Yes	35	22	13	
Postoperative sensory or motor dysfunction	No	53	20	33	0.110
	Yes	79	41	38	
Secondary ABC	No	74	33	41	0.674
	Yes	58	28	30	
Chicken-wire calcification	No	63	24	39	0.074
	Yes	69	37	23	
Recurrence during follow-up	No	95	39	56	0.057
	Yes	37	22	15	
S100	Low	27	13	14	0.821
	High	105	48	57	
Vim	Low	37	9	28	**0.002**
	High	95	52	43	
CK	Low	91	42	49	0.984
	High	41	19	22	

*ABC* aneurysmal bone cyst, *Vim* vimentin, *CK* cytokeratin

Bold values indicate *P* < 0.05

### Comparison of clinicopathological characteristics between ACB and EACB patients

When comparing the ACB and EACB cohorts, significant differences were observed between the two groups. Patients with ACB were found to be younger compared to those with EACB (*P* < 0.001, Table 1). Additionally, ACB patients had larger tumor sizes than EACB patients (*P* < 0.001, Table 1). Moreover, ACB patients exhibited a higher proportion of surrounding tissue invasion and a higher incidence of preoperative neuromotor dysfunction (*P* < 0.001 and *P* = 0.021, respectively, Table 1). Furthermore, ACB patients showed a higher frequency of high expression of vimentin (Vim) (*P* = 0.002, Table 1).

### Univariate Kaplan–Meier analysis and multivariate Cox analyses of prognostic factors in patients with ACB

**Fig. 3:**
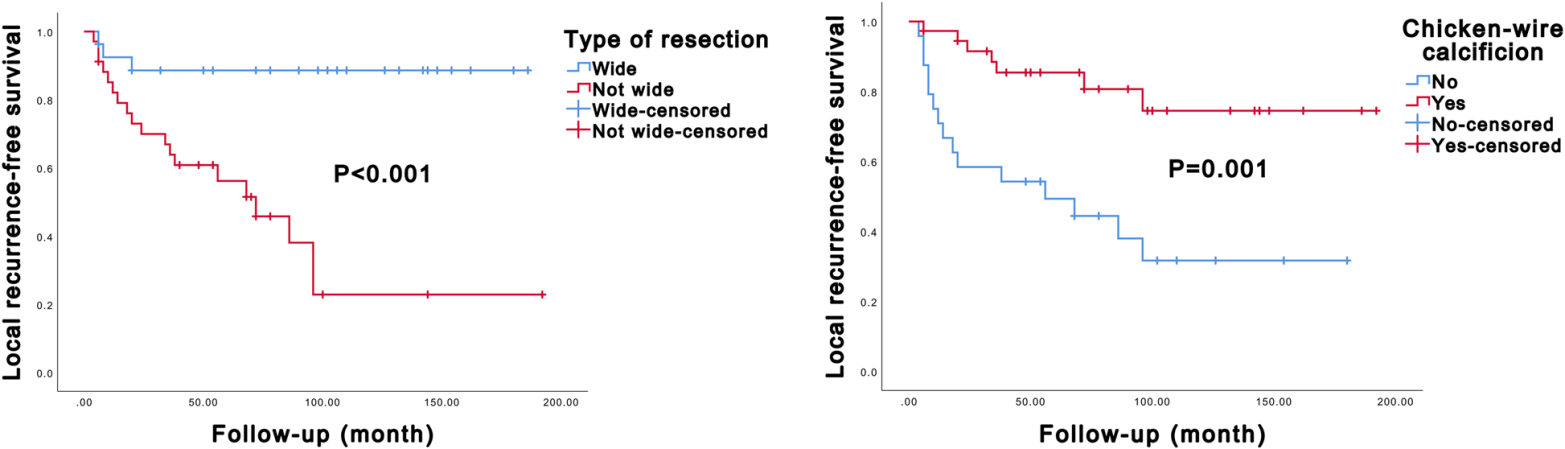
Kaplan–Meier curves of local recurrence-free survival of axial chondroblastoma patients stratified by type of resection and chicken-wire calcification

**Fig. 4:**
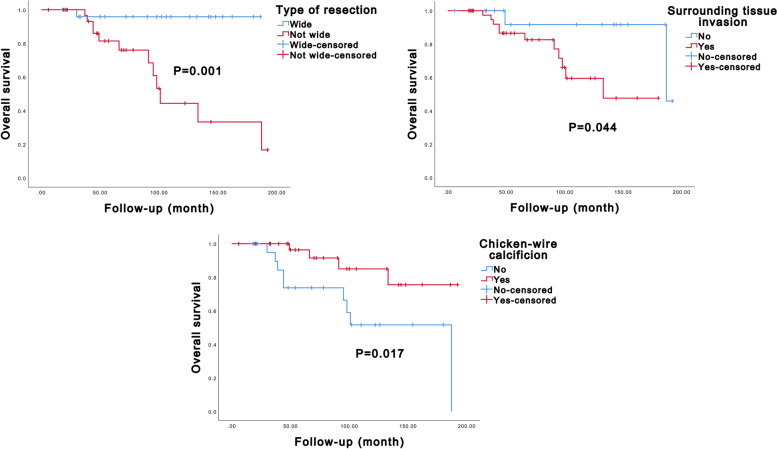
Kaplan–Meier curves of overall survival of axial chondroblastoma patients stratified by type of resection, surrounding tissue invasion, and chicken-wire calcification

The univariate Kaplan–Meier analysis revealed significant associations between several factors and LRFS and OS. Specifically, the type of resection and the presence of chicken-wire calcification were found to be significantly associated with LRFS (*P* < 0.001 and *P* = 0.001, respectively, Additional file 1: Supplemental Digital Content 3 and Fig. 3). Patients who underwent wide resection and exhibited chicken-wire calcification had better LRFS outcomes. Moreover, surrounding tissue invasion, type of resection, and chicken-wire calcification significantly influenced OS (*P* = 0.001, *P* = 0.044, and *P* = 0.017, respectively, Additional file 1: Supplemental Digital Content 3 and Fig. 4). Patients without surrounding tissue invasion, those who underwent wide resection, and those with chicken-wire calcification had longer OS, indicating a more favorable prognosis. Subsequently, in the multivariate Cox analysis, the type of resection and the presence of chicken-wire calcification emerged as independent predictors of LRFS (*P* = 0.002 and *P* = 0.003, respectively, Table 2), while the type of resection alone could independently predict OS (*P* = 0.027, Table 2).

**Table 2:** Multivariate Cox analyses of the prognostic factors of local recurrence-free survival and overall survival in patients with axial chondroblastoma

Factors	Categories	Numbers	Local recurrence-free survival		Overall survival	
			*P*-value	HR (95% CI)	*P*-value	HR (95% CI)
Type of resection	Wide	27	**0.002**	0.137 (0.039–0.479)	**0.027**	0.097 (0.012–0.768)
	Not wide	34				
Surrounding tissue invasion	No	18	/	/	0.192	0.242 (0.029**–**2.039)
	Yes	43				
Chicken-wire calcification	No	24	**0.003**	3.913 (1.583**–**9.672)	0.063	3.167 (0.938**–**10.695)
	Yes	37				

Bold values indicate *P* < 0.05

### Univariate Kaplan–Meier analysis and multivariate Cox analyses of prognostic factors in patients with EACB

**Fig. 5:**
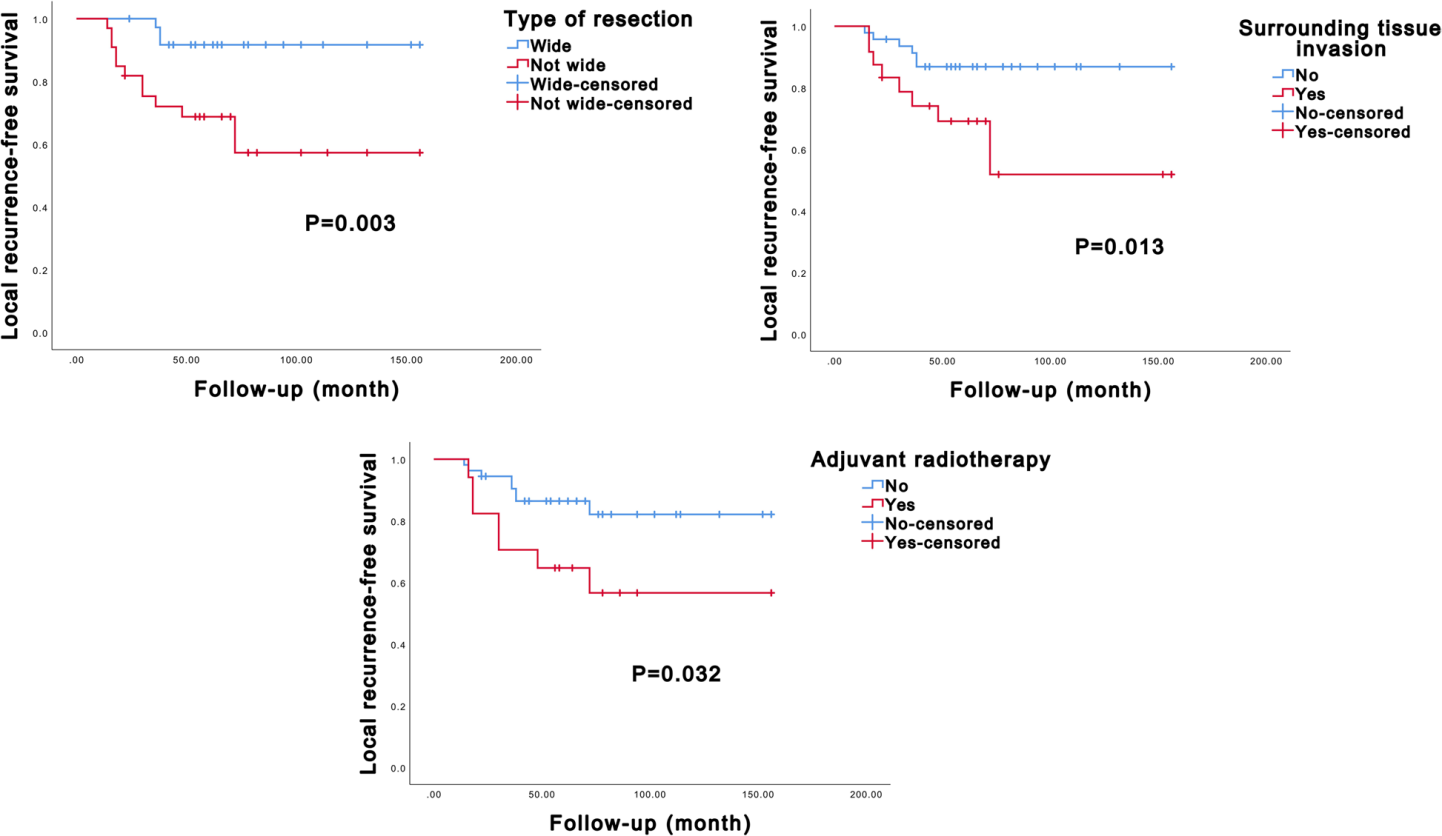
Kaplan–Meier curves of local recurrence-free survival of extra-axial chondroblastoma patients stratified by type of resection, surrounding tissue invasion, and adjuvant radiotherapy

**Fig. 6:**
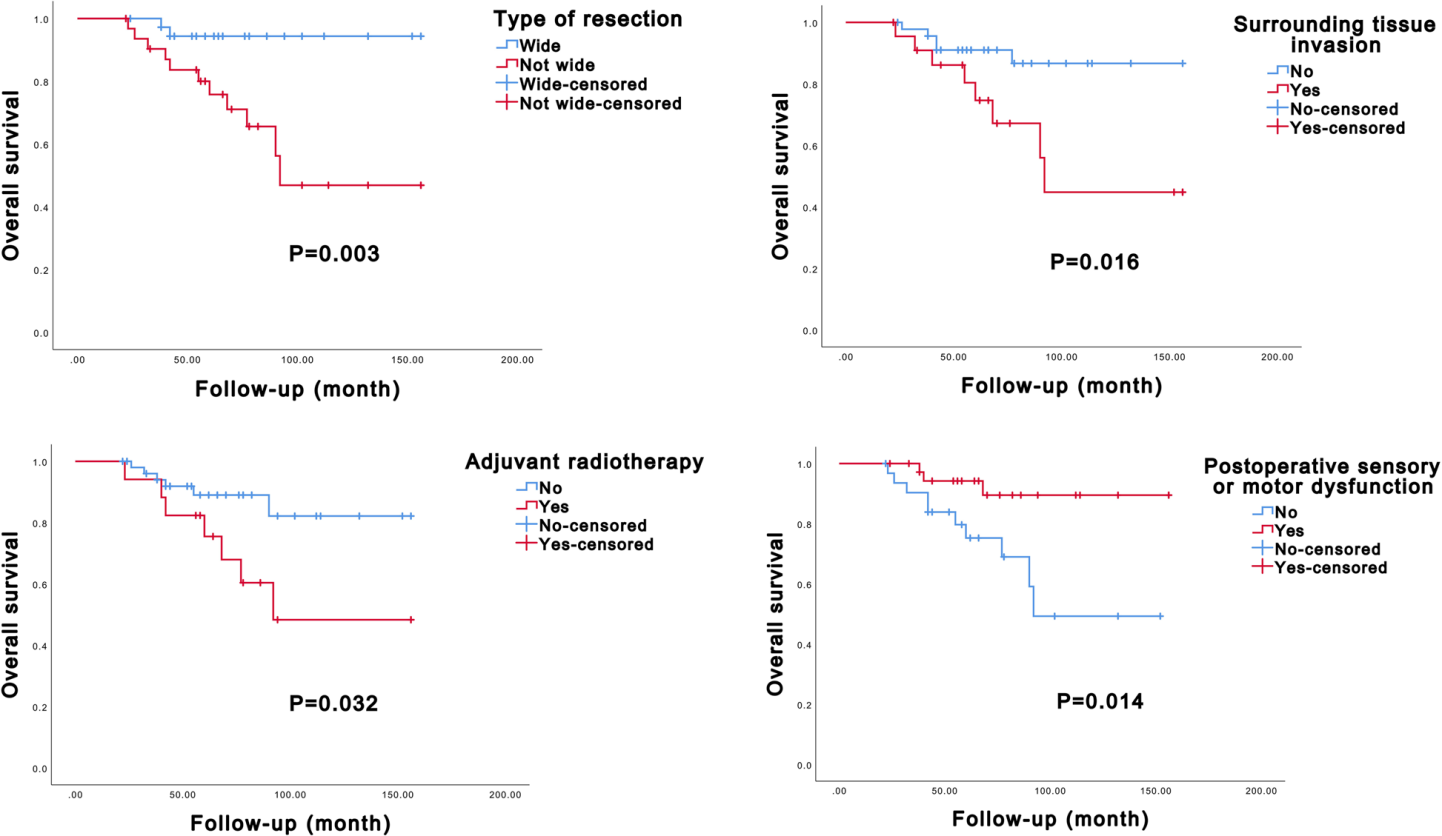
Kaplan–Meier curves of the overall survival of extra-axial chondroblastoma patients stratified by type of resection, surrounding tissue invasion, adjuvant radiotherapy, and postoperative sensory or motor dysfunction

The univariate Kaplan–Meier analysis showed that the type of resection, adjuvant radiotherapy, and surrounding tissue invasion were associated with LRFS (*P* = 0.003, *P* = 0.013, and *P* = 0.032, respectively, Additional file 1: Supplemental Digital Content 4 and Fig. 5). Patients who underwent wide resection had better LRFS, while those with surrounding tissue invasion and without adjuvant radiotherapy had poorer LRFS. Additionally, the type of resection, surrounding tissue invasion, adjuvant radiotherapy, and postoperative sensory or motor dysfunction were associated with patient OS (*P* = 0.003, *P* = 0.016, *P* = 0.014, and *P* = 0.014, respectively, Additional file 1: Supplemental Digital Content 4 and Fig. 6). Patients who underwent not-wide resection, had surrounding tissue invasion, did not receive radiotherapy, and experienced postoperative sensory or motor dysfunction had shorter OS, indicating a worse prognosis. In the multivariate Cox regression model, it was found that the type of resection and surrounding tissue invasion could independently predict LRFS (*P* = 0.019 and *P* = 0.041, respectively, Table 3), while the type of resection alone could independently predict OS (*P* = 0.039 and *P* = 0.032, respectively, Table 3).

**Table 3:** Multivariate Cox analyses of the prognostic factors of local recurrence-free survival and overall survival in patients with extra-axial chondroblastoma

Factors	Categories	Numbers	Local recurrence-free survival		Overall survival	
			*P*-value	HR (95% CI)	*P*-value	HR (95% CI)
Type of resection	Wide	27	**0.019**	4.774 (1.295–17.593)	**0.039**	5.405 (1.086–26.901)
	Not wide	34				
Surrounding tissue invasion	No	18	**0.041**	3.052 (1.048**–**8.889)	0.102	2.626 (0.826**–**8.344)
	Yes	43				
Adjuvant radiotherapy	No	54	0.313	1.739 (0.593**–**5.096)	0.365	1.728 (0.529**–**5.639)
	Yes	17				
Postoperative sensory or motor dysfunction	No	33	/	/	**0.032**	0.242 (0.066**–**0.887)
	Yes	38				

Bold values indicate *P* < 0.05

## Discussion

### Key results

In this study, we conducted a comprehensive analysis of the largest cohort of ACB patients and examined the relationship between clinicopathological characteristics and patient survival. Additionally, we compared the patient characteristics and prognostic factors between ACB and EACB patients. Our findings revealed several noteworthy observations. Firstly, ACB patients exhibited larger ages and tumor sizes compared to EACB patients. Furthermore, the incidence of surrounding tissue invasion and postoperative sensory or motor dysfunction was higher among ACB patients. Notably, high expression of Vim was more commonly observed in ACB patients. Regarding survival outcomes, the type of resection was associated with LRFS in both ACB and EACB cohorts, while the type of resection and surrounding tissue invasion were associated with OS. However, it is important to note that most other factors demonstrated inconsistent survival outcomes between the two groups. These findings suggest that ACB may possess distinct molecular and clinical characteristics compared to EACB. This comprehensive understanding of prognostic factors in ACB allows for the implementation of reasonable prognostic risk stratification, ultimately leading to improved patient survival.

### Differences in immunohistopathological and clinicopathological characteristics between ACB and EACB

This study aimed to compare the patient characteristics and prognostic patterns between ACB and EACB, shedding light on their biological differences. While most parameters exhibited similar expression in ACB and EACB, the expression of Vim was found to be higher in ACB. Vim is a major intermediate filament protein in mesenchymal cells and is associated with accelerated growth, infiltration, and poor prognosis in various cancers [18–21]. Based on this finding, we hypothesized that ACB may exhibit greater biological aggressiveness and a higher recurrence rate compared to EACB. This aligns with previous reports suggesting that spinal CB is more aggressive and prone to recurrence than bone CB in extremities [9–11]. Consistent with our hypothesis, we observed a larger tumor size and a higher incidence of surrounding tissue invasion in ACB patients, which indicate tumor aggressiveness and poor prognosis [22, 23]. Moreover, ACB patients were more likely to experience sensory or motor dysfunction, which can be attributed to the proximity of ACB tumors to neurovasculature in the spine and skull, increasing the risk of nerve damage compared to EACB. Additionally, we noted that the average age of ACB patients was higher than that of EACB patients. Interestingly, literature reports indicate that CB primarily affects individuals under 50 years of age, with a peak incidence in the 20–30 age range [5, 24], while cranial CB patients tend to be around 40 years old [7]. Furthermore, CB commonly occurs at the ends of long bones in young patients [4], whereas in older individuals, the tumor can arise at various sites, including the craniofacial skeleton [24, 25]. This could explain the higher average age observed in ACB patients. However, further comparative analysis with large sample data is warranted for future research.

### Influence of the type of resection and surrounding tissue invasion on the survival of ACB and EACB

Given the aggressive nature of CB, surgical intervention is considered essential for treatment [5, 10, 26]. Our study supports this notion by demonstrating that wide tumor resection leads to favorable LRFS outcomes in patients, consistent with previous findings [5, 10, 26]. It is widely recommended by experts to aim for the complete removal of tumor tissue during surgery to minimize the risk of postoperative recurrence and achieve optimal disease control [5, 10, 26]. This finding is further supported by a recent study specifically focusing on spinal CB [27]. Notably, it has been reported that the presence of residual lesions after surgery increases the likelihood of tumor recurrence [7]. Furthermore, our analysis revealed that patients with surrounding tissue invasion experienced shorter OS, which aligns with previous reports. This observation can be easily understood, as extensive tumor infiltration or the proximity of the tumor to critical nerves, blood vessels, and other tissues can hinder achieving wide resection during surgery, thereby increasing the likelihood of postoperative recurrence in patients [7, 27]. Additionally, it is worth noting that prolonged infiltration and tissue damage caused by tumor growth, as well as the potential impact of surgical intervention on important neurovascular structures and organs, may contribute to the worsening of symptoms and a subsequent decline in the patients’ anti-tumor immune function. These factors further create conditions that facilitate tumor recurrence and subsequently lead to an increased recurrence rate [28–30].

### Influence of chicken-wire calcification on the survival of ACB patients

Chicken-wire calcification is a commonly observed feature in the eosinophilic mechanism of CB and can serve as a diagnostic tissue marker for CB [5, 31, 32]. Our study aligns with a previous integrated analysis, demonstrating that spinal CB patients expressing chicken-wire calcification have a more favorable prognosis [17]. Previous research has shown that patients with calcification in tumor tissue experience significantly longer median progression-free survival and overall survival compared to those without calcification [33]. Calcification primarily involves the deposition of calcium salts and minerals, and bone-bridging proteins play a role in regulating this process [34]. On the other hand, osteopontin has been found to promote malignant tumor invasion, growth, and metastasis [35]. Thus, we hypothesize that the downregulation of osteopontin expression in ACB tumors may contribute to their reduced aggressiveness. Interestingly, studies have even demonstrated that among different types of calcification present in CB tumor tissue, patients with chicken-wire calcification have a better prognosis than those with non-chicken-wire calcification [36]. This difference may be attributed to distinct biological behaviors resulting from the spatial arrangements of calcifications, warranting further investigation into these theories.

### Influence of adjuvant radiotherapy on the survival of EACB

Another significant finding of this study revealed that EACB patients who underwent adjuvant radiotherapy had a poorer prognosis. This observation aligns with previous reports suggesting that radiotherapy may lead to the transformation of CB into a more malignant sarcoma [2, 7, 27]. In fact, some studies have even indicated that any form of adjuvant therapy should be avoided in CB patients [37]. On the contrary, radiotherapy has been shown to reduce tumor recurrence rates and can be beneficial for patients with postoperative recurrence or those deemed inoperable, leading to a favorable prognosis [9]. Consequently, the prognostic role of radiotherapy in CB remains controversial, and future studies involving larger sample sizes and detailed information on patient radiotherapy, combined with in vivo and in vitro experiments, are necessary to comprehensively evaluate the effects of adjuvant radiotherapy in CB patients. Current studies propose that radiation promotes epithelial-mesenchymal transition and induces the generation of new cancer stem cells from non-stem cells in various human cancers [38, 39]. This concept could serve as a theoretical foundation for the adverse prognosis observed in CB patients receiving adjuvant radiotherapy. Therefore, the detection and proteomic study of newly formed cancer stem cells may aid in identifying the precise mechanisms underlying disease progression in these CB patients.

In addition, numerous other factors significantly influence the prognosis of tumor patients, including preoperative frailty [40]. Frailty represents one of the most pressing global public health challenges, characterized by an accelerated decline in functional reserve associated with aging, rendering individuals more vulnerable following surgical procedures [41]. As a preoperative evaluation index, frailty has demonstrated remarkable prognostic prediction ability and risk stratification potential [41–43]. Studies have confirmed the correlation between frailty and an elevated risk of complications, as well as increased postoperative mortality. Timely identification of frail patients allows for the mitigation of vulnerable areas, thereby reducing the occurrence of complications and promoting favorable outcomes [44]. Therefore, based on our research findings, we recommend the implementation of frailty assessments in a timely and accurate manner for CB patients requiring surgical treatment. Integrating this research variable in the future can effectively assist CB patients in avoiding potential risks and enhance our ability to guide their clinical management more effectively.

### Limitation

Although this study is retrospective in nature, it is important to acknowledge the need for future prospective studies with larger sample sizes and comprehensive data records to further validate the findings presented here.

## Conclusion

This study aimed to provide a comprehensive analysis of clinicopathological characteristics and prognostic factors in a large cohort of ACB patients, as well as compare the differences in patient characteristics and prognostic patterns between ACB and EACB. Our findings revealed that Significant differences were observed between ACB and EACB patients in terms of age, tumor size, surrounding tissue invasion, preoperative sensory or motor dysfunction, and Vim expression. Notably, the impact of resection type and surrounding tissue invasion on prognosis remained consistent across both groups, while ACB and EACB exhibited distinct prognostic influences in other aspects. These results indicate the potential existence of diverse molecular and clinical behaviors between ACB and EACB, underscoring the significance of risk stratification and optimized treatment strategies for ACB patients.

## Supplementary Information

**Additional file 1:** **Supplemental Digital Content 1. **Determined cutoff values for age, duration of symptoms and tumor size in prognosis analysis of overall survival in axial chondroblastoma patients. DOS, duration of symptoms. **Supplemental Digital Content 2. **Determined cutoff values for age, duration of symptoms and tumor size in prognosis analysis of overall survival in extra-axial chondroblastoma. DOS, duration of symptoms. **Supplemental Digital Content 3. **Univariate analysis of the prognostic factors of localre currence-free survival and overall survival in patients with axial chondroblastoma. Bold values indicate *P* < 0.05;ABC, aneurysmal bone cyst; Vim, Vimentin; CK, cytokeratin. ^a^Cutoff points for patient age, duration of symptoms, tumor size in the survival analysis of OS were 35, 4.0, 5.0, respectively; ^b^P value from the log-rank test was corrected as previously suggested. **Supplemental Digital Content 4. **Univariate analysis of the prognostic factors of local recurrence-free survival and overall survival in patients with extra-axial chondroblastoma. Bold values indicate *P*< 0.05; ABC, aneurysmal bone cyst; Vim, Vimentin; CK, cytokeratin. ^a^Cutoffpoints for patient age, duration of symptoms, tumor size in the survival analysis of OS were 22, 5.0, 2.0, respectively; ^b^P value from the log-rank test was corrected as previously suggested.

## Data Availability

The datasets generated during and/or analyzed during the current study are available from the corresponding author upon reasonable request.
