# Supplementary material for: Clinicopathological characteristics and prognostic factors in axial chondroblastomas: a retrospective analysis of 61 cases and comparison with extra-axial chondroblastomas
Source: World J Surg Oncol. 2023 Jun 21;21:188. doi: 10.1186/s12957-023-03063-0 (PMC10283327; doi:10.1186/s12957-023-03063-0)
Supplement: Supplementary file 1 — Additional file 1: Supplemental Digital Content 1. Determined cutoff values for age, duration of symptoms and tumor size in prognosis analysis of overall survival in axial chondroblastoma patients. DOS, duration of symptoms. Supplemental Digital Content 2. Determined cutoff values for age, duration of symptoms and tumor size in prognosis analysis of overall survival in extra-axial chondroblastoma. DOS, duration of symptoms. Supplemental Digital Content 3. Univariate analysis of the prognostic factors of localre currence-free survival and overall survival in patients with axial chondroblastoma. Bold values indicate P < 0.05;ABC, aneurysmal bone cyst; Vim, Vimentin; CK, cytokeratin. aCutoff points for patient age, duration of symptoms, tumor size in the survival analysis of OS were 35, 4.0, 5.0, respectively; bP value from the log-rank test was corrected as previously suggested. Supplemental Digital Content 4. Univariate analysis of the prognostic factors of local recurrence-free survival and overall survival in patients with extra-axial chondroblastoma. Bold values indicate P< 0.05; ABC, aneurysmal bone cyst; Vim, Vimentin; CK, cytokeratin. aCutoffpoints for patient age, duration of symptoms, tumor size in the survival analysis of OS were 22, 5.0, 2.0, respectively; bP value from the log-rank test was corrected as previously suggested. [file 12957_2023_3063_MOESM1_ESM.doc]

**Supplemental Digital Content 1** Determined cutoff values for age, duration of symptoms and tumor size in prognosis analysis of overall survival in axial chondroblastoma patients.

**
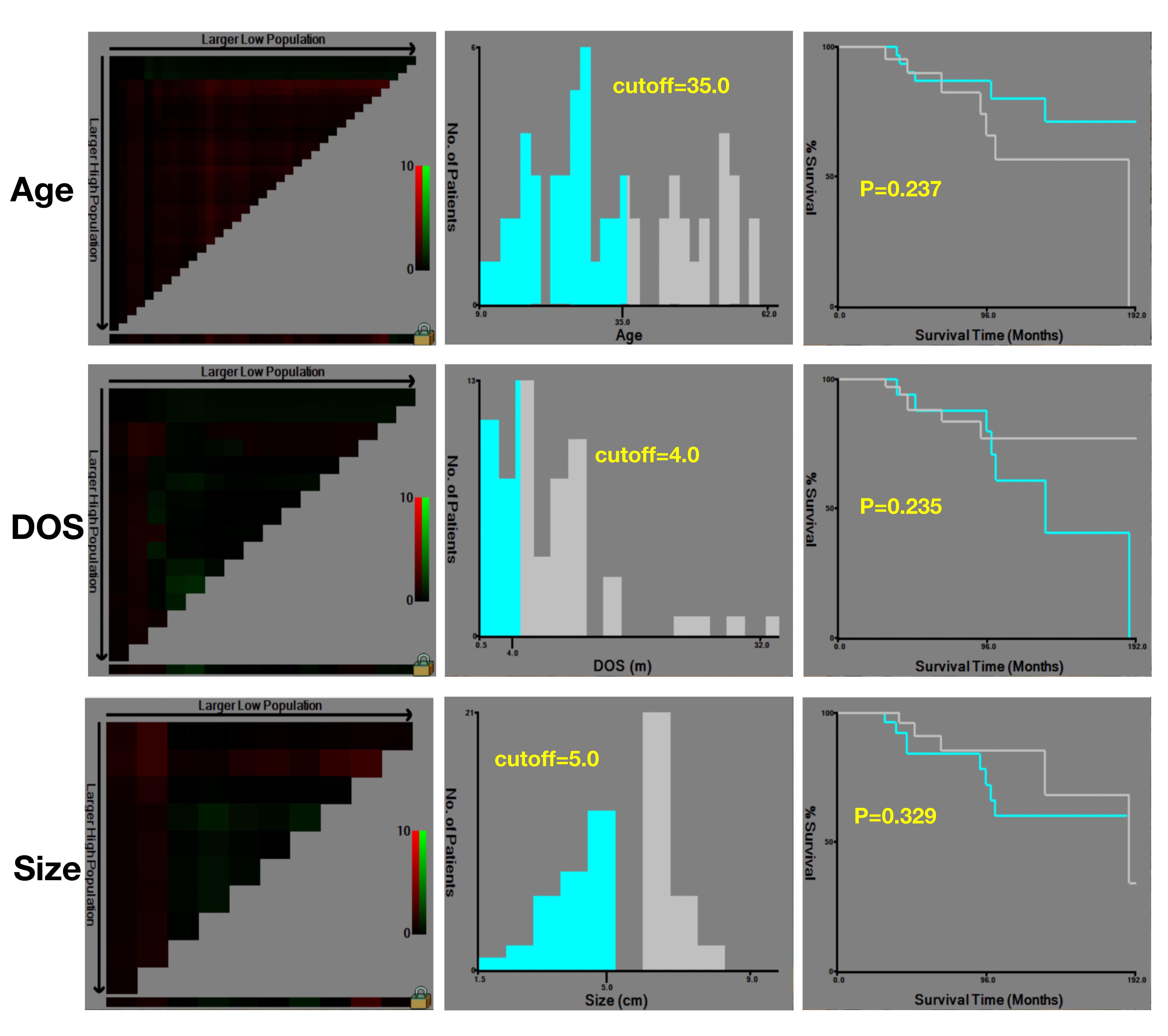
**

DOS, duration of symptoms.

**Supplemental Digital Content 2** Determined cutoff values for age, duration of symptoms and tumor size in prognosis analysis of overall survival in extra-axial chondroblastoma.

**
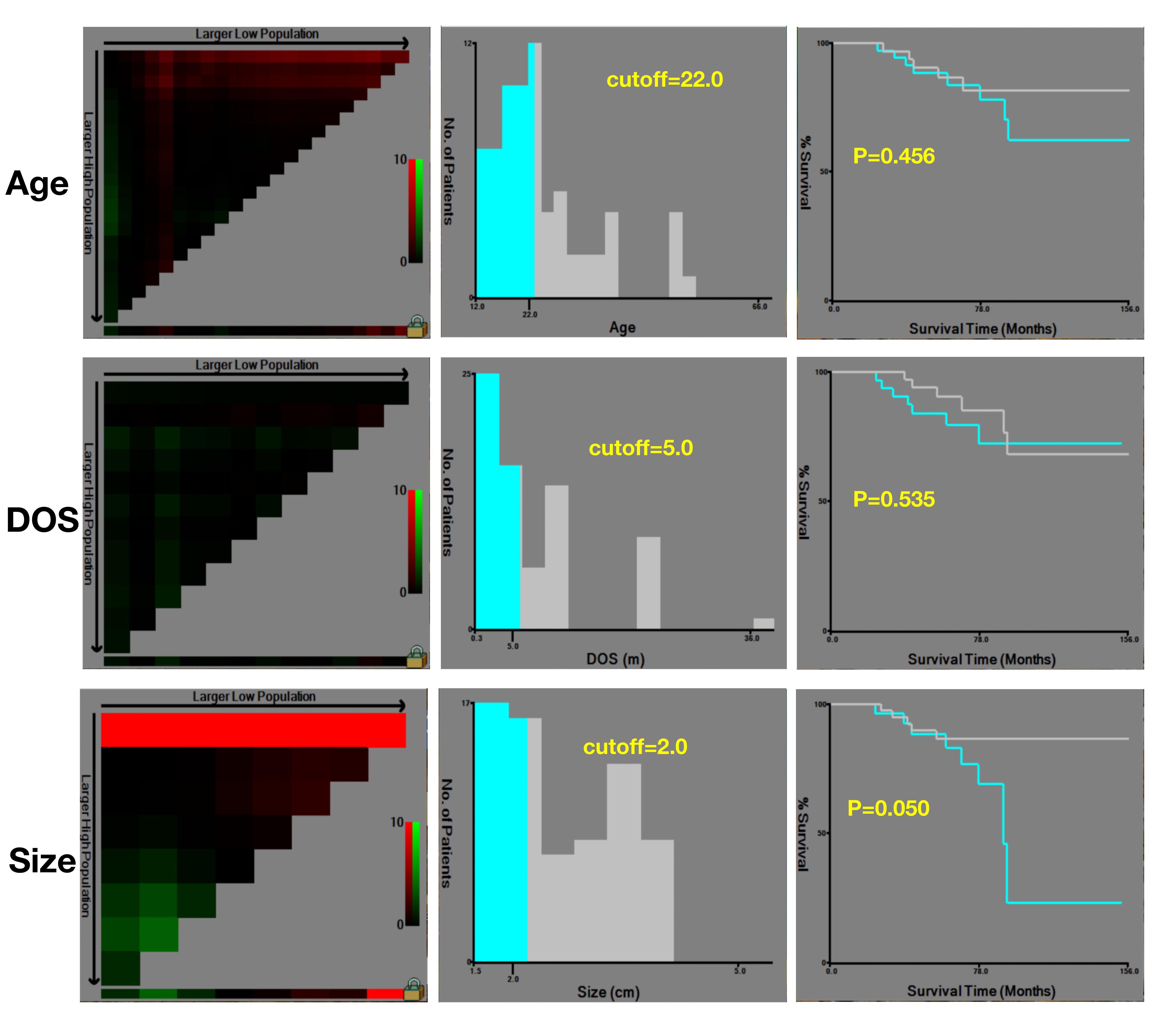
**

DOS, duration of symptoms.

**Supplemental Digital Content 3.** Univariate analysis of the prognostic factors of local recurrence-free survival and overall survival in patients with axial chondroblastoma

| Factors | Categories | Numbers | local recurrence-free survival | | overall survival | |
| --- | --- | --- | --- | --- | --- | --- |
|  |  |  | χ^2^ | *P*-value | χ^2^ | *P*-value |
| Age (years) | Young (≤ cutoff^a^) | 37 | 0.428 | 0.513^b^ | 1.399 | 0.237^b^ |
|  | Old (> cutoff^a^) | 24 |  |  |  |  |
| Gender | Female | 19 | 1.602 | 0.206 | 2.985 | 0.084 |
|  | Male | 42 |  |  |  |  |
| Duration of symptoms (months) | Short (≤ cutoff^a^) | 19 | 0.437 | 0.509^b^ | 1.409 | 0.235^b^ |
|  | Long (> cutoff^a^) | 42 |  |  |  |  |
| Tumor size (in diameter, cm) | Small (≤ cutoff^a^) | 30 | 0.001 | 0.980^b^ | 0.955 | 0.329^b^ |
|  | Large (> cutoff^a^) | 31 |  |  |  |  |
| Type of resection | Wide | 27 | 13.795 | **< 0.001** | 10.278 | **0.001** |
|  | Not wide | 34 |  |  |  |  |
| Surrounding tissue invasion | No | 18 | 3.675 | 0.055 | 4.041 | **0.044** |
|  | Yes | 43 |  |  |  |  |
| Adjuvant radiotherapy | No | 44 | 1.640 | 0.205 | 0.853 | 0.356 |
|  | Yes | 17 |  |  |  |  |
| Preoperative sensory or motor dysfunction | No | 39 | 0.099 | 0.753 | 0.853 | 0.356 |
|  | Yes | 22 |  |  |  |  |
| Postoperative sensory or motor dysfunction | No | 20 | 0.989 | 0.320 | 2.195 | 0.138 |
|  | Yes | 41 |  |  |  |  |
| Secondary ABC | No | 33 | 2.636 | 0.104 | 0.156 | 0.693 |
|  | Yes | 28 |  |  |  |  |
| Chicken-wire calcification | No | 24 | 11.416 | **0.001** | 5.679 | **0.017** |
|  | Yes | 37 |  |  |  |  |
| Tumoral S100 expression | Low | 13 | 0.946 | 0.331 | 0.151 | 0.698 |
|  | High | 48 |  |  |  |  |
| Tumoral Vim expression | Low | 9 | 0.109 | 0.741 | 0.001 | 0.974 |
|  | High | 52 |  |  |  |  |
| Tumoral CK expression | Low | 42 | 2.920 | 0.088 | 0.668 | 0.414 |
|  | High | 19 |  |  |  |  |

Bold values indicate *P* < 0.05; ABC, aneurysmal bone cyst; Vim, Vimentin; CK, cytokeratin . ^a^Cutoff points for patient age, duration of symptoms, tumor size in the survival analysis of OS were 35 , 4.0, 5.0, respectively; ^b^P value from the log-rank test was corrected as previously suggested.

**Supplemental Digital Content 4.** Univariate analysis of the prognostic factors of local recurrence-free survival and overall survival in patients with extra-axial chondroblastoma

| Factors | Categories | Numbers | local recurrence-free survival | | overall survival | |
| --- | --- | --- | --- | --- | --- | --- |
|  |  |  | χ^2^ | *P*-value | χ^2^ | *P*-value |
| Age (years) | Young (≤ cutoff^a^) | 37 | 0.415 | 0.519^b^ | 0.555 | 0.456^b^ |
|  | Old (> cutoff^a^) | 34 |  |  |  |  |
| Gender | Female | 27 | 2.006 | 0.157 | 0.931 | 0.335 |
|  | Male | 44 |  |  |  |  |
| Duration of symptoms (months) | Short (≤ cutoff^a^) | 32 | 0.725 | 0.391^b^ | 0.385 | 0.535^b^ |
|  | Long (> cutoff^a^) | 39 |  |  |  |  |
| Tumor size (in diameter, cm) | Small (≤ cutoff^a^) | 27 | 3.097 | 0.078^b^ | 3.838 | 0.050^b^ |
|  | Large (> cutoff^a^) | 44 |  |  |  |  |
| Type of resection | Wide | 38 | 8.837 | **0.003** | 8.583 | **0.003** |
|  | Not wide | 33 |  |  |  |  |
| Surrounding tissue invasion | No | 47 | 6.201 | **0.013** | 5.804 | **0.016** |
|  | Yes | 24 |  |  |  |  |
| Adjuvant radiotherapy | No | 54 | 4.616 | **0.032** | 4.588 | **0.032** |
|  | Yes | 17 |  |  |  |  |
| Preoperative sensory or motor dysfunction | No | 58 | 0.050 | 0.823 | 0.007 | 0.934 |
|  | Yes | 13 |  |  |  |  |
| Postoperative sensory or motor dysfunction | No | 33 | 3.415 | 0.065 | 6.054 | **0.014** |
|  | Yes | 38 |  |  |  |  |
| Secondary ABC | No | 41 | 0.123 | 0.725 | 0.002 | 0.964 |
|  | Yes | 30 |  |  |  |  |
| Chicken-wire calcification | No | 39 | 2.305 | 0.129 | 1.552 | 0.213 |
|  | Yes | 23 |  |  |  |  |
| Tumoral S100 expression | Low | 14 | 0.001 | 0.969 | 0.001 | 0.979 |
|  | High | 57 |  |  |  |  |
| Tumoral Vim expression | Low | 28 | 1.243 | 0.265 | 1.218 | 0.270 |
|  | High | 43 |  |  |  |  |
| Tumoral CK expression | Low | 49 | 2.846 | 0.092 | 1.865 | 0.172 |
|  | High | 22 |  |  |  |  |

Bold values indicate *P* < 0.05; ABC, aneurysmal bone cyst; Vim, Vimentin; CK, cytokeratin. ^a^Cutoff points for patient age, duration of symptoms, tumor size in the survival analysis of OS were 22, 5.0, 2.0, respectively; ^b^P value from the log-rank test was corrected as previously suggested.
